# Prostate cancer recurrence in the urethra with low PSA^☆^

**DOI:** 10.1016/j.eucr.2024.102787

**Published:** 2024-07-03

**Authors:** Shravankrishna Ananthapadmanabhan, Zoe Williams, Henry Wang, Alexander Combes, Veronica Wong, Isaac Thangasamy

**Affiliations:** a Nepean Urology Research Group, Nepean Hospital, Kingswood, New South Wales, 2747, Australia; b Department of Nuclear Medicine and PET, Nepean Hospital, Kingswood, New South Wales, 2747, Australia; c University of Sydney, Faculty of Medicine, Sydney, New South Wales, Australia

## Abstract

When localised prostate cancer recurs after treatment, it occurs predictably in sites such as the prostatic bed, pelvic lymph nodes, spine, lung, and liver. Urethral metastasis of prostate cancer is exceedingly rare. We report a case of urethral recurrence of prostate cancer presenting as new lower urinary tract symptoms in an 82-year-old male 10 years after robotic radical prostatectomy with a very low PSA level of 0.05μg/L. This rare case highlights the need to maintain a degree of suspicion for prostate cancer recurrence in patients with a late onset of or changing lower urinary tract symptoms after radical prostatectomy.

## 1 Introduction

Prostate cancer is the second most commonly diagnosed cancer in Australian.^1^ Management is dependent on disease grade and stage, patient's age and comorbidities, and patient preferences. Standard of care for localised, clinically significant prostate cancer includes Radical Prostatectomy, and Radiotherapy often supplemented with Androgen Deprivation Therapy (ADT).^2^

For patients who undergo surgical intervention, post-operative Prostate Sensitive Antigen (PSA) can be used initially as a marker of residual disease, and subsequently as a marker for disease recurrence or metastasis. Ideally, PSA levels should become undetectable after surgery. Rising PSA following an initial period of undetectable PSA signifies biochemical recurrence and may be indicative of local or distant cancer recurrence. In these settings, disease is often found in the prostatic bed, pelvic lymph nodes, and sites of distant metastases such as the spine, lung, and liver.^3^ Disease recurrence within the urethra is an exceedingly rare entity in the literature.

In this report, we present the case of an 82-year-old male who was diagnosed with metastatic prostatic adenocarcinoma in the anterior urethra, following investigation for lower urinary tract symptoms. His urological background includes Intermediate Risk Prostate Cancer which was treated with Robotic Assisted Radical Prostatectomy (RARP) over a decade ago. A literature review was performed on this uncommon site of metastasis, and the available evidence was synthesised and consolidated to inform management strategies and patient outcomes.

## 2 Case presentation

An 82-year-old male was reviewed by a urologist following onset of new voiding lower urinary tract symptoms (LUTS). His urological background was significant for clinically localised Intermediate Risk Prostate Cancer diagnosed in 2013 with a PSA of 10.0ug/L at the time. This was managed with a Robotic Radical Prostatectomy. Final histopathology confirmed International Society of Urological Pathology (ISUP) Grade Group 2 Prostate Acinar Adenocarcinoma. In the early post-operative years, PSA values were undetectable, but was observed to slowly increase to 0.03 μg/L in 2018. He was lost to follow-up in the intervening period between 2018 and 2023, and when representing for review of LUTS in 2023, was subsequently found to have a PSA of 0.05μg/L.

**Fig. 1:**
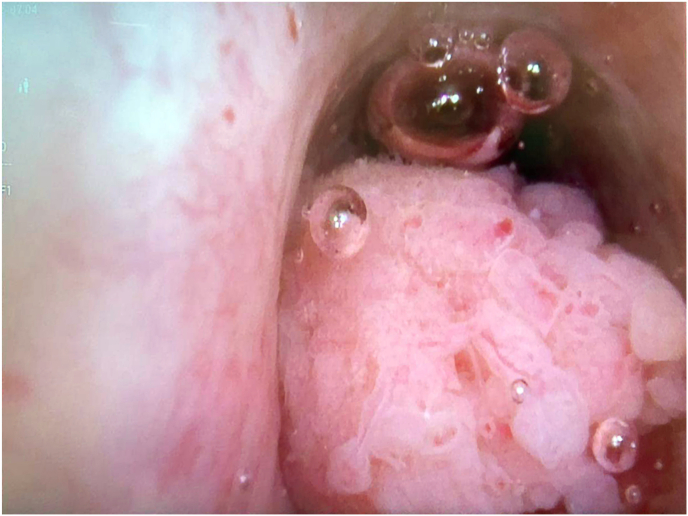
Cystoscopic view of anterior urethral tumour occluding 75 % of the urethral lumen.

While detectable on ultra-sensitive PSA assay, the PSA of 0.05μg/L did not meet the usual criteria for PSMA-PET of 0.2ug/L. His LUTS were investigated by cystoscopy which revealed a moderate sized anterior urethral lesion encompassing an estimated 75 % of the lumen (Fig. 1). There was no anastomotic stricture at the bladder neck, and the bladder demonstrated mild trabeculations. Although suspected to be urothelial carcinoma based on visual appearance, histopathology was instead demonstrative of ISUP Grade Group 4 Prostatic Acinar Adenocarcinoma.

A staging PSMA PET-CT was performed demonstrating moderate reactive PSMA uptake in the urethra at the biopsy site. No additional areas of PSMA-avidity were seen. Follow-up PSA at 12 weeks returned to undetectable levels (<0.01μg/L). The patient is undergoing routine cystoscopic surveillance and PSA monitoring. Lower urinary tract symptoms resolved following urethral lesion clearance.

## 3 Discussion

Following treatment of localised prostate cancer, serial measurement of prostate specific antigen (PSA) forms the cornerstone of detecting cancer recurrence.^2^ PSA is a serine protease produced by prostatic tissue and is the most sensitive marker of prostate cancer recurrence following local therapy.^4^

Prostate cancer recurrence following radical prostatectomy recurs predictably in sites such as the prostatic bed, pelvic lymph nodes, spine, lung, and liver.^3^ Metastasis to the urinary tract is exceedingly rare with our literature review yielding fewer than 25 cases reported.^5^, ^6^, ^7^, ^8^, ^9^, ^10^, ^11^, ^12^, ^13^ In almost all cases, the primary symptom observed at time of recurrence was gross haematuria, with isolated LUTS considerably less common. In cases where urethral metastases were preceded by prostate cancer treatment, time to recurrence ranged from 1 to 13 years following index treatment. Serum PSA at time of urethral metastases was reported as low as 0.13μg/L.

The mechanisms through which urethral metastases occur are not clearly understood. Cases of urethral metastasis have been reported in the setting of direct tumour extension from the prostate, however this may instead represent invasive primary disease. Additionally, the theory of retrograde lymphatic spread may not be anatomically sound, due to the anterior urethra's predilection to drain into the inguinal nodes. Other theories hypothesised include implantation after instrumentation and hematogenous spread via arterial tumour emboli or retrograde venous dissemination. In our literature review, the majority of cases were associated with prior urinary tract instrumentation or surgery supporting the hypothesis of direct implantation post instrumentation.^5^, ^6^, ^7^^,^^10^, ^11^, ^12^, ^13^ We hypothesise that this is also the most likely aetiology in our case.

Numerous treatments have been employed in the management of urethral metastases, with transurethral resection being the most common. Additionally, Cystoprostatectomy with urethrectomy, chemotherapy, radiotherapy have also been reported. In our case, cold-cup biopsy was chosen, as the size of the tumours facilitated complete macroscopic clearance without need for loop resection and the associated risks of extensive urethral or corporal injury. The case was discussed at a multi-disciplinary meeting, and as the PSA returned to undetectable levels, the recommendation was to proceed with continued endoscopic and PSA surveillance.

Long-term survival data for treatments of isolated urethral metastasis is limited. Kasai et al. reported a case where radiotherapy was utilised, with no recurrence at 50 months’ follow-up.^11^ Wang et al. reported a case of ductal metastasis to the urethra managed with endoscopic resection. Recurrence was seen on repeat cystoscopy at 12 months with further resection performed.^9^

This case highlights the importance of maintaining an index of suspicion and need to perform endoscopic work-up for late onset of LUTS following RARP. While these symptoms are more commonly associated with underlying urinary tract infections, bladder neck anastomotic strictures, or urethral strictures, they also harbour the rare possibility of prostate cancer metastasis to the urethra.

## 4 Conclusion

We report a case of metastatic prostate cancer involving the urethra in the setting of new LUTS, on a background of previous RARP for clinically localised Intermediate Risk Prostate cancer. An index of suspicion should be maintained for disease recurrence, even in the setting of minimally elevated PSA levels. Macroscopic urethral clearance by biopsy resolved the LUTS with return of PSA to undetectable levels.

## Statement of ethics

Informed patient consent has been obtained for publication of this case report and clinical images.

## CRediT authorship contribution statement

**Shravankrishna Ananthapadmanabhan:** Writing – original draft. **Zoe Williams:** Writing – review & editing. **Henry Wang:** Writing – review & editing, Conceptualization. **Alexander Combes:** Writing – review & editing. **Veronica Wong:** Writing – review & editing. **Isaac Thangasamy:** Supervision, Conceptualization.

## Declaration of competing interest

The authors have no conflicts of interest to declare.
